# Proportion of venous thromboembolism attributed to recognized prothrombotic genotypes in men and women

**DOI:** 10.1016/j.rpth.2024.102343

**Published:** 2024-02-08

**Authors:** Carl Arne Løchen Arnesen, Line H. Evensen, Kristian Hveem, Maiken E. Gabrielsen, John-Bjarne Hansen, Sigrid K. Brækkan

**Affiliations:** 1 Thrombosis Research Group, Department of Clinical Medicine, UiT–The Arctic University of Norway, Tromsø, Norway; 2 Thrombosis Research Center, Division of Internal Medicine, University Hospital of North Norway, Tromsø, Norway; 3 HUNT Center for Molecular and Clinical Epidemiology, Department of Public Health and Nursing, Norwegian University of Science and Technology, Trondheim, Norway; 4 HUNT Research Center, Department of Public Health and Nursing, Norwegian University of Science and Technology, Levanger, Norway; 5 Levanger Hospital, Nord-Trøndelag Hospital Trust, Levanger, Norway

**Keywords:** epidemiology, risk factors, sex differences, single-nucleotide polymorphism, venous thromboembolism

## Abstract

**Background:**

Data on the proportion of venous thromboembolism (VTE) risk attributed to prothrombotic genotypes in men and women are limited.

**Objectives:**

We aimed to estimate the population attributable fraction (PAF) of VTE for recognized, common prothrombotic genotypes in men and women using a population-based case cohort.

**Methods:**

Cases with incident VTE (*n* = 1493) and a randomly sampled subcohort (*n* = 13,069) were derived from the Tromsø study (1994-2012) and the Trøndelag Health Study (1995-2008) cohorts. DNA samples were genotyped for 17 single-nucleotide polymorphisms (SNPs) previously associated with VTE. PAFs with 95% bias-corrected CIs (based on 10,000 bootstrap samples) were estimated for SNPs significantly associated with VTE, and a 6-SNP cumulative model was constructed for both sexes.

**Results:**

In women, the individual PAFs for SNPs included in the cumulative model were 16.9% for *ABO* (rs8176719), 17.6% for *F11* (rs2036914), 15.1% for *F11* (rs2289252), 8.7% for *FVL* (rs6025), 6.0% for *FGG* (rs2066865), and 0.2% for *F2* (rs1799963). The cumulative PAF for this 6-SNP model was 37.8%. In men, the individual PAFs for SNPs included in the cumulative model were 21.3% for *ABO*, 12.2% for *F11* (rs2036914), 10.4% for *F11* (rs2289252), 7.5% for *FVL*, 7.8% for *FGG*, and 1.1% for *F2*. This resulted in a cumulative PAF in men of 51.9%.

**Conclusion:**

Our findings in a Norwegian population suggest that 52% and 38% of the VTEs can be attributed to known prothrombotic genotypes in men and women, respectively.

## Essentials

• Sex-specific data on venous thrombosis risk attributed to prothrombotic genotypes are scarce.
• We estimated the cumulative population attributable risk of 6 single-nucleotide polymorphisms in men and women.
• For this 6-single-nucleotide polymorphism model, the cumulative population attributable risk was 51.9% in men and 37.8% in women.
• Prothrombotic genotypes appeared to explain a higher proportion of venous thromboses in men than in women.

## 1 Introduction

Venous thromboembolism (VTE) is a common and complex disease that is the result of both genetic and environmental factors [1]. The risk of VTE in men vs women has been shown to vary according to age, with a higher risk in women than in men among the young (<50 years) and a higher risk in men than in women among the middle-aged (50-75 years) [2,3]. Even though the overall lifetime risk of VTE appears to be similar in men and women [4], the contribution of different risk factors to disease development may vary across both sex and age.

VTE has a strong hereditary component, and family and twin studies have suggested an overall VTE heritability of 45% to 60% [[5], [6], [7], [8], [9]]. Of note, some family studies suggest a stronger genetic component in men than in women [9], whereas others find little [7] or no overall difference [5]. Several single-nucleotide polymorphisms (SNPs) associated with increased VTE risk have been detected [[10], [11], [12], [13], [14]], and genome-wide association studies (GWASs) report that the recognized prothrombotic SNPs contribute 15% to 20% of the VTE heritability [15]. Few studies have investigated the impact of the recently identified SNPs on VTE risk in men and women separately. Moreover, the proportion of the VTEs that can be attributed to these SNPs in men and women in different age groups has not been studied in a general population.

Population attributable fraction (PAF) estimates the fraction of disease that potentially can be prevented if efficient interventions are available [16,17]. From an epidemiological perspective, PAF is a useful tool to quantify disease risk attributable to genetic factors. We have previously shown that a 6-SNP cumulative PAF model explained about 45% of all VTEs in the population [18]. Whether the individual and combined PAFs of known prothrombotic SNPs for VTE are similar in men and women has not been addressed. Additionally, whether genetic risk impacts men and women differently in certain age groups has not been extensively studied. Therefore, this study aimed to estimate (i) the individual and cumulative PAF of established prothrombotic SNPs in men and women and (ii) the cumulative PAF in men and women across age groups in a population-based case cohort.

## 2 Methods

### 2.1 Study population

The population under study was derived from the fourth survey of the Tromsø study (Tromsø 4) and the second survey of the Trøndelag Health Study (HUNT). Both studies are population-based cohort studies of residents in the Tromsø municipality and Nord-Trøndelag County, respectively. To Tromsø 4 (1994/1995), all residents aged at least 25 years were invited, and 77% (27,158) participated. To the second survey of HUNT (1995-1997), all residents aged at least 20 years were invited, and 71% (66,140) participated. A thorough description of the Tromsø [19] and HUNT [20] studies can be found in their cohort profile papers.

Follow-up of participants started from the inclusion date in the respective cohort surveys and lasted until the date of incident VTE, migration, death, or end of follow-up (December 31, 2012, in Tromsø and December 31, 2008, in HUNT), whichever occurred first. The procedures for identification of all VTEs in the Tromsø study [21] and the HUNT Study [22] have been extensively presented elsewhere and will be briefly summarized here. For the Tromsø study, information on VTE events was obtained by searching the radiology procedure registry, the autopsy registry, and the hospital discharge diagnosis registry at the University Hospital of North Norway. The medical documents of each patient were carefully examined. The adjudication criteria for VTE were symptoms and signs of pulmonary embolism or deep vein thrombosis, followed by objective verification by radiological imaging and initiation of anticoagulant treatment (unless treatment contraindications were present). In the HUNT Study, identification of VTE events was obtained by searching the radiology procedure registry and the discharge diagnosis registry at the 2 local hospitals, Namsos and Levanger, as well as the discharge diagnosis registry at St. Olavs Hospital in Trondheim, the tertiary care center of the region. Two doctors examined the medical records, and all radiologically verified VTE events among the participants were recorded.

**Figure 1:**
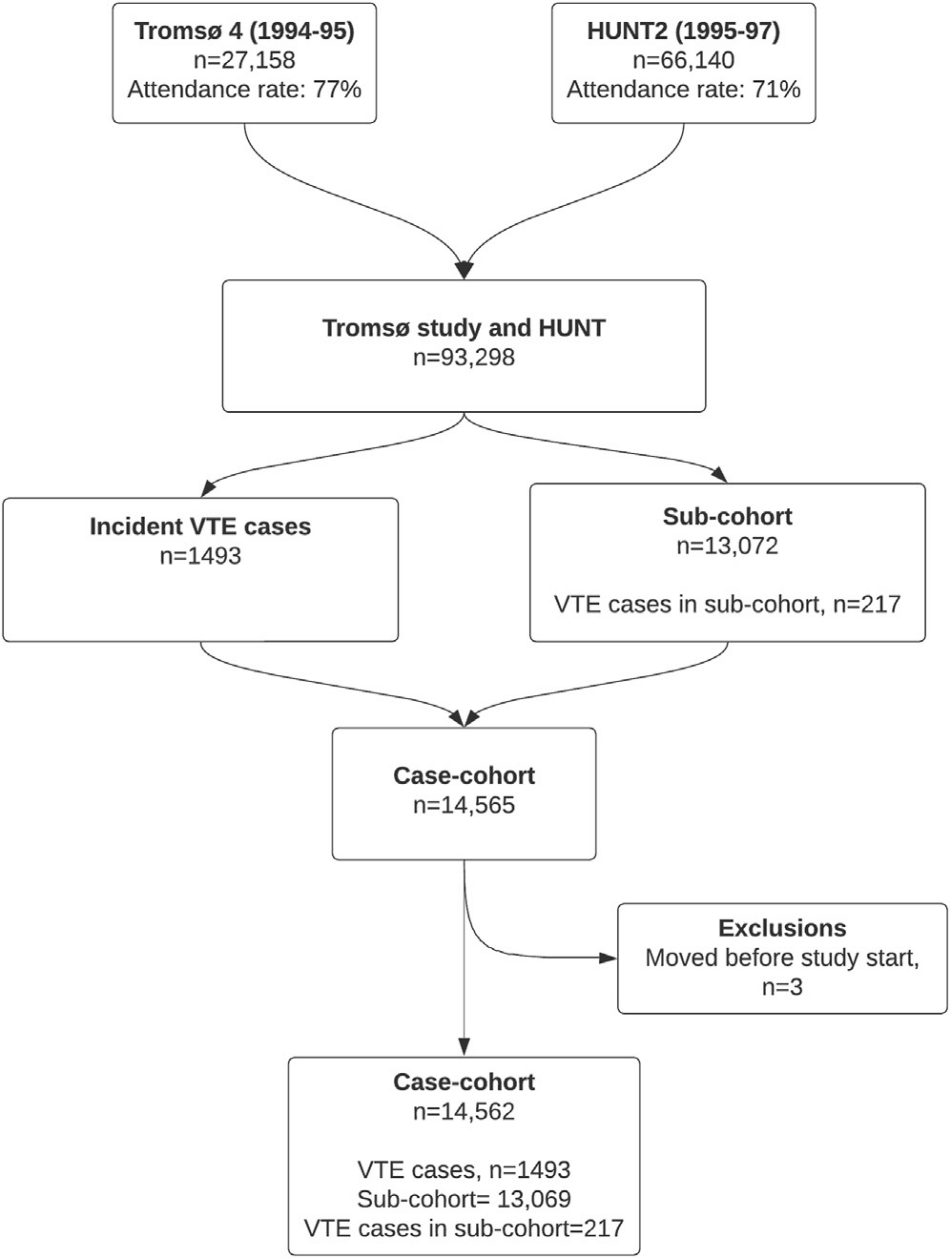
Overview of the case cohort study composition. HUNT, Trøndelag Health Study; HUNT2, second survey of the Trøndelag Health Study; Tromsø 4, fourth survey of the Tromsø Study; VTE, venous thromboembolism.

A case cohort was created based on all cases with a first-lifetime VTE (*n* = 1493) and a subcohort (*n* = 13,072) randomly sampled from the source cohorts (Tromsø and HUNT). Participants in the source cohorts with a known VTE event before attending the baseline survey were excluded before the creation of the case cohort. Participants not registered as residents of Tromsø or Nord-Trøndelag at study inclusion (*n* = 3) were excluded. Ultimately, the case cohort consisted of 14,562 participants (1493 VTE cases and 13,069 subcohort participants). The composition of the case cohort is displayed in Figure 1. When sampling the subcohort, every person in the source cohort (including cases) has the same probability of being sampled. Therefore, 217 of the VTE cases were by chance also sampled to the subcohort. The Regional Committee of Medical Health Research Ethics approved the study, and all study participants gave their informed written consent to participate.

### 2.2 Baseline measurements

Baseline information was collected by physical examinations, blood samples, and self-administered questionnaires in both studies. Information on arterial cardiovascular disease (stroke, myocardial infarction, and angina) and diabetes was self-reported. Measurement of weight and body height was conducted with participants wearing no shoes and light clothing, and body mass index (BMI) was calculated.

### 2.3 SNP selections and genotyping

Genotyping was performed for 17 SNPs with recognized association with VTE risk. The TaqMan (Thermo Fisher) and Sequenom platforms (Sequenom) were used for genotyping in the Tromsø study, as previously documented [10], while the Illumina HumanCore Exome array was used for genotyping in HUNT.

Participants were categorized as carriers (≥1 risk allele), or noncarriers (0 risk alleles), and no distinction was made between hetero- and homozygous carriers in the PAF and hazard ratio (HR) analyses. For the SNPs rs2036914 (*F11*), rs1801020 (*F12*), rs1039084 (*STXBP5*), rs1884841 (*TC2N*), rs1613662 (*GP6*), and rs4524 (*F5*), the major alleles were labeled as the risk alleles [[11], [12], [13],^23^]. For the rs8176719 SNP (in *ABO*), the G allele marks non-O blood type (risk group), while those who were homozygous carriers for deletion at this site were classified as blood type O and served as the reference group [23].

### 2.4 Statistical analysis

Stata version 16 (Stata Corp) was used to run the statistical analyses. The person-time of follow-up was accumulated from the date of inclusion for each participant until the date of first-time VTE, migration, death, or the end of follow-up. Follow-up ended on December 31, 2012, in Tromsø and on December 31, 2008, in HUNT. All analyses were run distinctly for women and men. For each SNP, sex-specific HRs for VTE with 95% CIs were estimated using Cox regression models with noncarriers (0 risk alleles) serving as reference category. Age served as a time scale. The entry time was the age at inclusion, and the exit time was the age at VTE/censoring. The HR model was adjusted for BMI. Schoenfeld residuals were used to evaluate the proportional hazards assumption, which was not violated.

PAF was calculated for each SNP using the formula $\frac{p\left(\text{HR}-1\right)}{p\left(\text{HR}-1\right)+1}$, where *HR* is the VTE risk in carriers vs noncarriers of the risk allele, and *p* is the prevalence of carriers in the population [16]. We applied a cumulative PAF model previously created by Evensen et al. [18]. The model was constructed by adding SNPs one-by-one in order based on the highest individual PAF estimates, beginning with the 2 SNPs with the highest estimates in the total population. Despite a low PAF, prothrombin (*F2*) was added to the model due to its considerable HR and because it is a well-recognized risk factor for VTE. The SNPs included in this cumulative PAF model were *ABO* (rs8176719), *F11* (rs2036914), *F11* (rs2289252), *FVL* (rs6025), *FGG* (rs2066865), and *F2* (rs1799963). The model was applied to men and women separately. The calculation of 95% bias-corrected CIs for the PAF estimates was based on 10,000 bootstrap samples. We also calculated the cumulative PAF of the 6-SNP model according to separate age groups (≤50, 50-75, and >75 years) in men and women. We omitted individuals with missing information on SNPs included in the cumulative PAF model (women, *n* = 28; men, *n* = 87) and BMI (women, *n* = 66; men, *n* = 25) from these analyses (to preserve identical samples in each analysis).

## 3 Results

Descriptive information on VTE cases and the subcohort according to sex are displayed in Table 1. Mean age, systolic blood pressure, BMI, diabetes, and the proportion of arterial cardiovascular diseases were higher among the VTE cases compared with the subcohort in both sexes.

**Table 1:** Baseline characteristics of venous thromboembolism cases and the subcohort.

	VTE cases (*n* = 1493)		Subcohort (*n* = 13,069)	
	Women (*n* = 790)	Men (*n* = 703)	Women (*n* = 6909)	Men (*n* = 6160)
Age (y), mean ± SD	62 ± 15	60 ± 13	51 ± 17	50 ± 16
BMI (kg/m^2^), mean ± SD	27.9 ± 5.0	26.9 ± 3.6	26.2 ± 4.6	26.3 ± 3.5
Systolic blood pressure (mm Hg), mean ± SD	145.9 ± 25.0	143.8 ± 20.8	136.4 ± 24.2	140.1 ± 18.6
Arterial cardiovascular disease,^a^ % (*n*)	11.8 (93)	16.2 (114)	6.1 (420)	10.1 (619)
Diabetes, % (*n*)	4.1 (32)	4.6 (32)	3.1 (217)	3.1 (193)
History of cancer, % (*n*)	7.2 (57)	5.5 (39)	4.1 (280)	3.1 (193)

BMI, body mass index; VTE, venous thromboembolism.

a Myocardial infarction, stroke, and angina.

Sex-specific allele frequencies and distribution of risk alleles for all prothrombotic SNPs in the cases and the subcohort are shown in Table 2. In the subcohort, the allele frequencies were essentially similar in men and women and comparable to those reported in other populations of European ancestry [24].

**Table 2:** Sex-specific allele frequency and distribution of risk alleles in venous thromboembolism cases and the subcohort in descending order based on allele frequency in the subcohort.

Gene	SNP	VTE cases (*n* = 1493)						Subcohort (*n* = 13,069)					
		Women (*n* = 790)			Men (*n* = 703)			Women (*n* = 6909)			Men (*n* = 6160)		
		AF (%)	1 risk allele, % (*n*)	2 risk alleles, % (*n*)	AF (%)	1 risk allele, % (*n*)	2 risk alleles, % (*n*)	AF (%)	1 risk allele, % (*n*)	2 risk alleles, % (*n*)	AF (%)	1 risk allele, % (*n*)	2 risk alleles, % (*n*)
*GP6*	rs1613662	85.0	24.2 (191)	72.9 (576)	87.1	22.1 (155)	76.1 (534)	82.7	29.0 (2005)	68.2 (4708)	82.5	28.3 (1744)	68.4 (4209)
*F12*	rs1801020	76.5	35.1 (228)	58.9 (383)	76.1	34.6 (203)	58.8 (345)	74.6	37.2 (2517)	56.0 (3788)	74.2	38.9 (2348)	54.7 (3304)
*F5*	rs4524	75.8	36.0 (284)	57.9 (457)	77.6	37.5 (263)	58.8 (413)	72.8	39.2 (2706)	53.2 (3677)	72.9	39.3 (2416)	53.2 (3277)
*F11*	rs2036914	58.1	51.1 (404)	32.5 (257)	56.4	47.9 (335)	32.4 (227)	53.3	50.4 (3479)	28.1 (1940)	52.8	49.9 (3054)	27.9 (1706)
*STXBP5*	rs1039084	53.9	48.4 (382)	29.8 (235)	53.6	47.9 (336)	29.6 (208)	51.3	50.4 (3482)	26.1 (1800)	51.7	50.4 (3100)	26.5 (1631)
*TC2N*	rs1884841	45.4	53.6 (422)	18.6 (146)	43.5	47.4 (333)	19.8 (139)	43.2	48.1 (3319)	19.2 (1323)	42.9	48.4 (2980)	18.6 (1148)
*KNG1*	rs710446	43.2	48.2 (381)	19.1 (151)	40.8	44.7 (314)	18.5 (130)	41.5	48.9 (3379)	17.0 (1176)	41.0	47.9 (2947)	17.0 (1048)
*F11*	rs2289252	45.3	48.4 (382)	21.1 (167)	41.3	49.2 (345)	16.7 (117)	39.3	47.5 (3275)	15.6 (1077)	39.0	47.3 (2913)	15.3 (944)
*ABO*	rs8176719	42.7	50.6 (400)	17.3 (137)	43.6	52.0 (364)	17.6 (123)	38.3	45.9 (3166)	15.3 (1056)	38.4	46.7 (2861)	15.1 (922)
*VWF*	rs1063857	37.8	49.8 (377)	13.0 (98)	37.8	46.6 (318)	14.5 (99)	38.3	46.4 (2878)	15.0 (932)	38.0	46.8 (2611)	14.6 (812)
*F13*	rs5985	29.2	39.4 (253)	9.5 (61)	28.3	41.8 (243)	7.4 (43)	27.2	40.0 (2692)	7.2 (484)	26.1	38.2 (2291)	7.1 (423)
*FGG*	rs2066865	26.6	35.6 (281)	8.9 (70)	28.0	39.5 (278)	8.3 (58)	23.6	36.1 (2845)	5.6 (386)	24.3	36.6 (2255)	6.0 (370)
*SERP*	rs2227589	10.4	18.5 (146)	9 (1.1)	9.0	15.8 (111)	1.1 (8)	8.8	15.8 (1091)	0.9 (59)	8.7	15.9 (978)	0.8 (48)
*C4BPB*	rs3813948	7.8	14.6 (115)	0.5 (4)	7.0	12.8 (90)	0.6 (4)	7.7	14.3 (986)	0.6 (39)	7.6	14.0 (862)	0.6 (39)
*FVL*	rs6025	7.9	15.0 (118)	0.4 (3)	7.6	13.7 (96)	0.7 (5)	3.3	6.3 (434)	0.1 (10)	3.5	6.8 (420)	0.1 (5)
*F2*	rs3136520	3.0	5.8 (45)	0.1 (1)	3.3	6.0 (42)	0.3 (2)	3.1	6.0 (413)	0.1 (7)	3.0	5.7 (350)	0.2 (9)
*F2*	rs1799963	0.8	1.5 (12)	-	1.4	2.7 (19)	-	0.6	1.2 (85)	-	0.7	1.4 (88)	-

AF, allele frequency; SNP, single-nucleotide polymorphism; VTE, venous thromboembolism.

Table 3 contains the sex-specific HRs and PAF estimates with 95% CIs of VTE for all 17 individual SNPs. As displayed in Table 3, the HRs and PAFs of the individual SNPs diverged somewhat between the sexes. The most noticeable differences in PAFs were found for rs1884841 (*TC2N*: 11.6%; 95% CI, −1.3% to 24.4% in women vs 2.1%; 95% CI, −11.8% to 15.9% in men), rs2036914 (*F11*: 17.6%; 95% CI, 0.4%-34.1% for women vs 12.2%; 95% CI, −5.5% to 29.2% for men), rs8176719 (*ABO*: 16.9%; 95% CI, 5.8%-28.0% for women vs 21.3%; 95% CI, 9.6%-32.5% for men), and rs2289252 (*F11*: 15.1%; 95% CI, 3.4%-26.7% for women vs 10.4%; 95% CI, −2.0% to 22.4% for men).

**Table 3:** Hazard ratio and population attributable fraction with 95% CIs for venous thromboembolism by individual single-nucleotide polymorphisms in men and women in alphabetical order.

Gene	SNP	Women			Men		
		Subcohort prevalence^a^	HR (95% CI)^b^	PAF (95% CI)	Subcohort prevalence^a^	HR (95% CI)^b^	PAF (95% CI)
*ABO*	rs8176719	0.61	1.33 (1.15 to 1.55)	16.9 (5.8 to 28.0)	0.62	1.44 (1.23 to 1.69)	21.3 (9.6 to 32.5)
*C4BPB*	rs3813948	0.15	1.01 (0.83 to 1.23)	0.2 (−3.5 to 4.2)	0.15	0.93 (0.75 to 1.16)	−1.0 (−4.8 to 2.9)
*FGG*	rs2066865	0.42	1.15 (1.00 to 1.33)	6.0 (−1.8 to 14.2)	0.43	1.20 (1.03 to 1.39)	7.8 (−0.5 to 16.0)
*FVL*	rs6025	0.06	2.48 (2.04 to 3.00)	8.7 (5.4 to 12.3)	0.07	2.17 (1.76 to 2.69)	7.5 (4.0 to 11.2)
*F2*	rs1799963	0.01	1.17 (0.65 to 2.13)	0.2 (−0.8 to 1.5)	0.01	1.79 (1.13 to 2.81)	1.1 (−0.3 to 2.9)
*F2*	rs3136520	0.06	0.95 (0.70 to 1.28)	−0.3 (−2.5 to 2.1)	0.06	1.16 (0.85 to 1.57)	0.9 (−1.6 to 3.7)
*F5*	rs4524	0.92	1.27 (0.95 to 1.70)	20.0 (−10.9 to 47.3)	0.92	1.97 (1.33 to 2.92)	47.3 (19.0 to 72.1)
*F11*	rs2036914	0.79	1.27 (1.05 to 1.54)	17.6 (0.4 to 34.1)	0.78	1.18 (0.98 to 1.42)	12.2 (−5.5 to 29.2)
*F11*	rs2289252	0.63	1.28 (1.10 to 1.49)	15.1 (3.4 to 26.7)	0.63	1.19 (1.01 to 1.39)	10.4 (−2.0 to 22.4)
*F12*	rs1801020	0.93	1.04 (0.75 to 1.44)	3.6 (−38.3 to 38.3)	0.94	0.89 (0.64 to 1.23)	−11.1 (−57.8 to 29.9)
*F13*	rs5985	0.47	1.07 (0.92 to 1.25)	3.3 (−6.2 to 2.1)	0.45	1.15 (0.97 to 1.35)	6.1 (−3.6 to 15.6)
*GP6*	rs1613662	0.97	0.9 (0.60 to 1.37)	−10.3 (−74.2 to 44.6)	0.97	1.62 (0.94 to 2.80)	37.5 (−10.2 to 76.4)
*KNG1*	rs710446	0.66	1.07 (0.92 to 1.24)	4.2 (−8.1 to 16.7)	0.65	0.97 (0.83 to 1.14)	−1.7 (−15.1 to 11.2)
*SERP*	rs2227589	0.17	1.20 (1.01 to 1.43)	3.2 (−0.9 to 7.7)	0.17	1.00 (0.80 to 1.20)	−0.2 (−4.4 to 4.1)
*STXBP5*	rs1039084	0.76	1.15 (0.97 to 1.37)	0.1 (−5.3 to 27.0)	0.77	1.03 (0.86 to 1.22)	1.9 (−15.9 to 18.8)
*TC2N*	rs1884841	0.67	1.20 (1.02 to 1.40)	11.6 (−1.3 to 24.4)	0.67	1.03 (0.88 to 1.20)	2.1 (−11.8 to 15.9)
*VWF*	rs1063857	0.61	1.05 (0.91 to 1.22)	2.7 (−7.5 to 13.6)	0.61	1.01 (0.87 to 1.18)	0.7 (−10.6 to 12.0)

HR, hazard ratio; PAF, population attributable fraction; SNP, single-nucleotide polymorphism.

a Great than or equal to the risk allele.

b Adjusted for age (as time scale) and body mass index.

**Figure 2:**
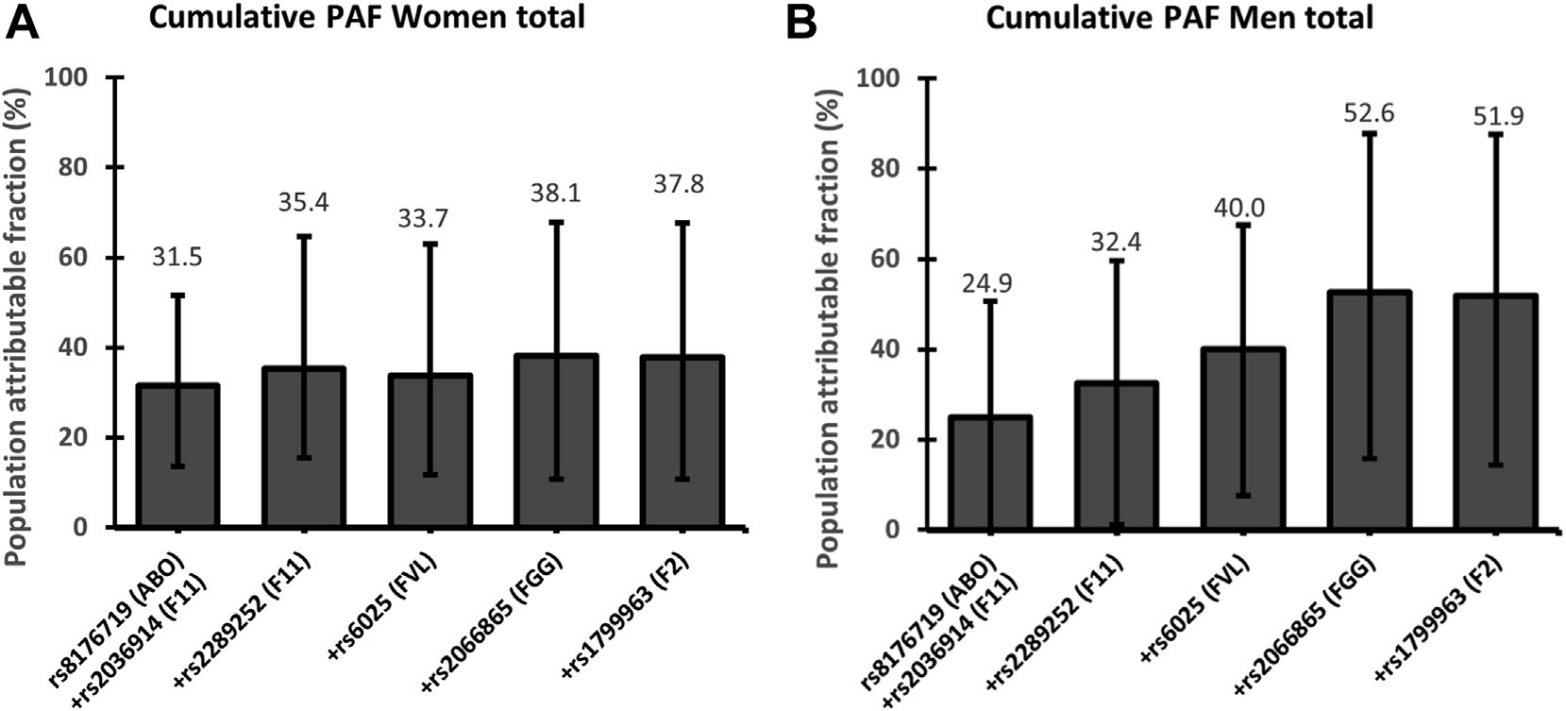
Cumulative population attributable fraction (PAF) of venous thromboembolism by increasing number of single-nucleotide polymorphisms (SNPs) in (A) women and (B) men. The following SNPs were included: rs8176719 (*ABO*), rs2036914 (*F11*), rs2289252 (*F11*), rs6025 (*FVL*), rs2066865 (*FGG*), and rs1799963 (*F2*). SNPs were added in order of the individual PAF estimates (Table 3).

**Figure 3:**
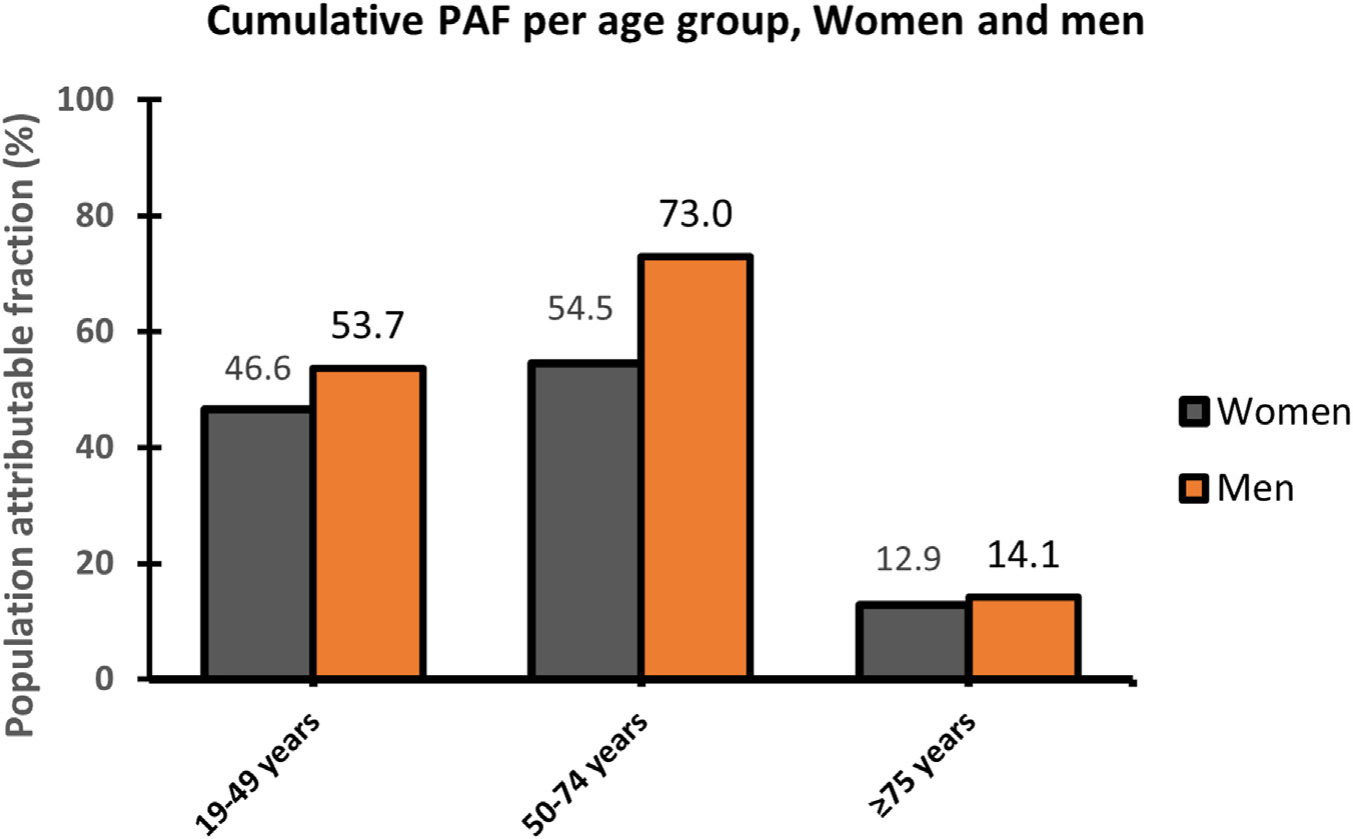
Cumulative population attributable fraction (PAF) of prothrombotic single-nucleotide polymorphisms (SNPs) stratified by age (<50, 50-74, and ≤75) for women and men. The following SNPs were included: rs8176719 (*ABO*), rs2036914 (*F11*), rs2289252 (*F11*), rs6025 (*FVL*), rs2066865 (*FGG*), and rs1799963 (*F2*). SNPs were added in order of the individual PAF estimates (Table 3).

Figure 2 shows the results of the previously established 6-SNP cumulative PAF model for women (Figure 2A) and men (Figure 2B) separately. In total, 51.9% of the VTE cases in the male population could be explained by these 6 SNPs, while the corresponding estimate in the female population was only 37.8%. The cumulative PAFs for the 6-SNP model according to age groups in men and women are shown in Figure 3. In those <50 years, the cumulative PAF was 46.6% and 53.7% in women and men, respectively, while the corresponding PAF in those aged 50 to 75 years was 54.5% (women) and 73.0% (men). In people >75 years, the cumulative PAF of the 6 SNPs was 12.9% in women and 14.1% in men.

## 4 Discussion

In the present study, we investigated the individual risk of VTE contributed by 17 known prothrombotic genotypes in men and women and estimated the total and age-specific cumulative PAF for the combination of 6 SNPs (*ABO*, *F11* [rs2289252], *F11* [rs2036914], *FVL*, *FGG*, and *F2*). Even though the frequencies of the 17 SNPs did not differ between men and women, the relative risk estimates and individual PAFs differed slightly for some of the SNPs. Furthermore, the fraction of VTEs that could be ascribed to the 6 prothrombotic SNPs was 38% in women and 52% in men. Analyses stratified into young, middle-aged, and elderly individuals showed cumulative PAFs of 54%, 73%, and 14% in men, respectively, while the corresponding PAFs in women were 47%, 54%, and 13%, respectively. Thus, particularly among the middle-aged, prothrombotic genotypes appeared to explain a higher proportion of the VTE events in men than in women.

Few studies have reported on the association between all 17 SNPs and the risk of VTE in men and women separately. Neither of these SNPs is linked to sex chromosomes, and as expected, the allele frequencies of the various SNPs were similar in men and women in our subcohort. Moreover, the allele frequencies in our study corresponded well with those reported in other Caucasian populations [[25], [26], [27]]. Using the Multiple environmental and genetic assessment of risk factors for venous thrombosis (MEGA) case-control study, Roach et al. [28] investigated the effect of *FVL*, prothrombin mutation, and non-O blood group on the risk of VTE in men and women separately. They reported similar odds ratios (ORs) and PAFs for both sexes, with individual PAFs in men and women of 10% and 8% for *FVL*, 4% and 3% for prothrombin, and 39% and 35% for non-O blood group, respectively. For prothrombin and the non-O blood group, the ORs and PAF estimates in MEGA were considerably higher than in our study. This may depend to some extent on the case-control design and the younger age distribution (and thereby lower baseline risk of VTE) of the MEGA participants. Of note, our risk estimates for *FVL*, *F11*, *FGG*, and *ABO* corresponded well with those reported in a GWAS meta-analysis of 18 studies [15]. We found that rs1884841 in *TC2N* was associated with increased VTE risk in women but not in men, resulting in PAF estimates of 11.6% in women and 2.1% in men. Previous studies on *TC2N* and VTE have shown somewhat diverging results. The study that first identified *TC2N* as a potential risk factor for VTE reported ORs ranging from 1.08 to 1.30 in 3 European case-control studies [11]. A later meta-analysis of GWAS data from 18 studies found a weak association with an OR of 1.05 [15]. Neither of these studies reported risk estimates for men and women separately. In contrast with our findings, 2 studies restricted to women [27,29] reported no association between *TC2N* and VTE (OR, 1.02). Thus, even though our results suggest that *TC2N* could serve as a substantial attributable genetic risk factor in women, we cannot rule out the possibility of a chance finding.

To our knowledge, no study has investigated the cumulative PAF of prothrombotic genotypes in men and women separately. In a case-control study, a joint PAF of 40% was reported for the SNPs in *F2* (rs1799963), *F5* (rs6025), *ABO* (rs2519093), and *ABO* (rs8176719) [26]. However, no sex-specific analyses were presented, and several of the SNPs included in our study were not investigated in their study [26]. We have previously shown that the model containing *ABO*, *F11*, *F11*, *FGG*, *FVL*, and *F2* yielded a cumulative PAF of 45% in the total population. However, our sex-specific analyses revealed that this model explained a larger proportion (52%) of the VTEs in men than in women (38%).

We found that the portion of VTE events attributable to prothrombotic genotypes varied in men and women of different ages. Few previous studies have investigated sex-specific genetic VTE risk in different age groups. In a cross-sectional study of patients aged >40 years with first-time VTE, Weingarz et al. [30] reported a higher prevalence of thrombophilia in men than in women. Furthermore, Zöller et al. [5] reported slightly higher standardized incidence ratios in men than in women in a nationwide study of siblings aged 20 to 60 years. Of note, the sex differences observed in these studies did not reach statistical significance. In a study of VTE cases and controls aged 18 to 70 years, Roach et al. [31] hypothesized that the observed higher VTE risk in men compared with women without reproductive risk factors could be explained by a genetic risk difference. However, further study of *FVL*, prothrombin, and *ABO*-blood group yielded only marginally higher risk estimates in men than in women in the same population [28]. In our study, there was a moderate difference in the cumulative PAF in individuals aged <50 years (women, 46.6%; men, 53.7%). In this age range, female reproductive risk factors such as pregnancy and oral contraceptives [4,8,28,32] are well-known contributors to VTE risk, and the impact of these strong risk factors may explain why a lower proportion of all VTEs were attributed to genetics in women. Among the middle-aged (50-75 years), the cumulative PAF of prothrombotic genotypes was substantially higher in men than in women in our study (women, 54.5%; men, 73.0%). Considering that the prevalence of reproductive risk factors in the middle-aged is low [33] and that the use of postmenopausal hormonal therapy has declined substantially in the Norwegian population in the last decades [34], it is unlikely that the PAF disparity in this age group is due to reproductive risk factors. A possible explanation could be that women who are genetically susceptible to VTE are affected at a younger age than men with a similar genetic profile due to the additive influence of reproductive risk factors. In addition, genetically susceptible men could be more vulnerable in middle age due to an additive effect from risk factors such as body height [35,36] and obesity [37]. This hypothesis is supported by the “catch-up” effect observed in men within this age range when modeling the cumulative lifetime risk in men and women separately [4]. Finally, the substantially lower PAF of prothrombotic genotypes observed in the elderly (women, 12.9%; men, 14.1%) could be explained by further depletion of susceptibles in both sexes, as well as the higher prevalence of other major VTE risk factors in this age group, such as cancer, surgery, and immobility [8]. This age pattern has previously been reported in both family history and genotype studies [5,18,30], further supporting the idea that people with a prothrombotic genotype usually experience VTE earlier in life, regardless of sex.

Our study used PAF to quantify the proportion of VTEs that could be attributed to known prothrombotic risk factors. The PAF estimate is usually larger than other genetic measures, like heritability [38], which quantifies the effects on the variability of risk at the population level. Although PAF and heritability measure different aspects of genetic contributions to disease risk, the 2 measurements are related [17]. While PAF is a useful tool to analyze the influence of genetic factors, it can be overestimated when the risk factor prevalence is very high due to its direct dependence on prevalence [38,39]. The complete, cumulative PAF models used in our study had a high prevalence, and consequently, the percentages might be somewhat overestimated. However, since the prevalence of the SNPs was similar in men and women, our PAF estimates would still allow for comparison between the sexes.

The main strengths of this study are its prospective design, high participation rate, large number of genotyped participants, and long-term follow-up in both the Tromsø and HUNT studies. Moreover, VTE events were objectively confirmed and systematically validated, and the genotyping had few missing values for most SNPs. The study cohort is a homogenous Caucasian population, which limits the likelihood of confounding by ethnicity but also limits its generalizability beyond this group. Due to the overlap between the present sample and the sample in which the associations were estimated, our results could be susceptible to overestimation of the genetic association, more commonly known as the winner’s curse [40]. To avoid this, we ensured that the SNPs selected for the cumulative PAF models corresponded well with previous observations from the literature [23,26,27,29] and refrained from selecting SNPs based solely on the HRs in our own sample. The statistical power for some subgroup analyses resulting in wide CIs for some of the risk estimates, especially affecting the bootstrap estimates for PAF, implies that these results should be interpreted with caution. We cannot rule out the possibility that some participants included in the subcohort could have had a previous VTE. Nevertheless, the prevalence of (misclassified) previous VTE is expected to be low, and if present, this would likely have a negligible impact on the risk estimates*.* Additionally, we did not run any subgroup analysis on the type of VTE, which perhaps could have revealed whether some SNPs are more relevant for specific types of VTE.

In conclusion, our findings in a Norwegian population suggest that 52% and 38% of the VTEs can be attributed to known, common prothrombotic genotypes in men and women, respectively. The proportion of VTE events that could be attributed to genotypes varied across age groups in both sexes and was particularly high among middle-aged men. In the elderly, the PAF of prothrombotic genotypes was similar in men and women.
